# Dbx1 Pre-Bötzinger Complex Interneurons Comprise the Core Inspiratory Oscillator for Breathing in Unanesthetized Adult Mice

**DOI:** 10.1523/ENEURO.0130-18.2018

**Published:** 2018-05-28

**Authors:** Nikolas C. Vann, Francis D. Pham, Kaitlyn E. Dorst, Christopher A. Del Negro

**Affiliations:** Department of Applied Science, Integrated Science Center, William & Mary, Williamsburg, VA 23185

**Keywords:** breathing, central pattern generator, Dbx1, pre-Bötzinger complex, respiration

## Abstract

The brainstem pre-Bötzinger complex (preBötC) generates inspiratory breathing rhythms, but which neurons comprise its rhythmogenic core? Dbx1-derived neurons may play the preeminent role in rhythm generation, an idea well founded at perinatal stages of development but incompletely evaluated in adulthood. We expressed archaerhodopsin or channelrhodopsin in Dbx1 preBötC neurons in intact adult mice to interrogate their function. Prolonged photoinhibition slowed down or stopped breathing, whereas prolonged photostimulation sped up breathing. Brief inspiratory-phase photoinhibition evoked the next breath earlier than expected, whereas brief expiratory-phase photoinhibition delayed the subsequent breath. Conversely, brief inspiratory-phase photostimulation increased inspiratory duration and delayed the subsequent breath, whereas brief expiratory-phase photostimulation evoked the next breath earlier than expected. Because they govern the frequency and precise timing of breaths in awake adult mice with sensorimotor feedback intact, Dbx1 preBötC neurons constitute an essential core component of the inspiratory oscillator, knowledge directly relevant to human health and physiology.

## Visual Abstract

**Figure:**
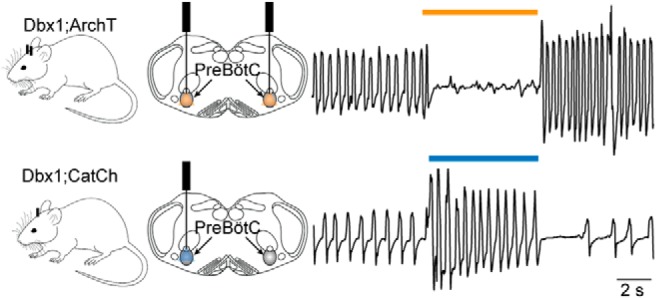

## Significance Statement

Breathing behavior depends on rhythmic movements. The underlying neural rhythm for inspiration may originate due to brainstem interneurons defined genetically by expression of the embryonic transcription factor Dbx1. Dbx1-derived neurons comprise the core oscillator microcircuit in perinatal mice, but they serve other functions too, and their inspiratory rhythmogenic role has not been conclusively tested in adults. Optogenetic photostimulation and photoinhibition of Dbx1-derived brainstem neurons in intact adult mice modulated breathing, either speeding it up, slowing it down to the point of apnea (no breathing), or perturbing its phase, which are functions consistent with the rhythm generator. These results establish the cellular point of origin for breathing rhythm, a key physiologic brain function in humans and all mammals.

## Introduction

Inspiratory breathing movements in mammals originate from neural rhythms in the brainstem pre-Bötzinger complex (preBötC; Smith et al., 1991; Feldman et al., 2013). Although the preBötC has been identified in a range of mammals including bats, moles, goats, cats, rabbits, rats, mice, and humans (Smith et al., 1991; Schwarzacher et al., 1995, 2011; Mutolo et al., 2002; Wenninger et al., 2004; Pantaleo et al., 2011; Ruangkittisakul et al., 2011; Tupal et al., 2014) its neuronal constituents remain imprecise. Competing classification schemes emphasize peptide and peptide receptor expression (Gray et al., 1999, 2001; Stornetta et al., 2003a; Tan et al., 2008) as well as a glutamatergic transmitter phenotype (Funk et al., 1993; Stornetta et al., 2003b; Wallen-Mackenzie et al., 2006) as cellular markers that define the preBötC rhythmogenic core.

Interneurons derived from precursors that express the homeodomain transcription factor Dbx1 (i.e., Dbx1 neurons) also express peptides and peptide receptors associated with respiratory rhythmogenesis and are predominantly glutamatergic. *Dbx1* knock-out mice die at birth of asphyxia and the preBötC never forms (Bouvier et al., 2010; Gray et al., 2010). In rhythmically active slice preparations from neonatal Dbx1 reporter mice, Dbx1 preBötC neurons discharge in bursts in phase with inspiration (Picardo et al., 2013), and their sequential laser ablation slows and then stops respiratory motor output (Wang et al., 2014). These results obtained from perinatal mice suggest that Dbx1 neurons comprise the rhythmogenic preBötC core; we refer to this idea as the Dbx1 core hypothesis.

Nevertheless, in addition to their putatively rhythmogenic role, Dbx1 preBötC neurons also govern motor pattern. Hypoglossal motoneurons that maintain airway patency receive rhythmic synaptic drive from Dbx1 neurons within the preBötC and adjacent intermediate reticular formation (Wang et al., 2014; Revill et al., 2015; Song et al., 2016). In anesthetized, vagotomized adult mice, photostimulation of Dbx1 preBötC neurons modulates inspiratory timing and its motor pattern, which is mediated in part by somatostatin-expressing (Sst) preBötC neurons (Cui et al., 2016), a large fraction of which are derived from Dbx1-expressing progenitors (Bouvier et al., 2010; Gray et al., 2010; Koizumi et al., 2016).

In adult animals, Dbx1 preBötC neurons serve non-respiratory roles as well. A subset that expresses Cadherin-9 (Cdh9) projects to the pontine locus coeruleus to influence arousal (Yackle et al., 2017). Collectively, the fractions of motor output-related (Sst-expressing) and arousal-related (Cdh9-expressing) Dbx1 neurons could account for 73% of Dbx1 neurons within the preBötC: up to 17% of Dbx1 preBötC neurons express Sst and 56% express Cdh9 with no overlap between Sst and Cdh9 expression (Bouvier et al., 2010; Gray et al., 2010; Cui et al., 2016; Yackle et al., 2017). That accounting would leave 27% of Dbx1 preBötC neurons exclusively rhythmogenic, if one assumes that all remaining Dbx1 neurons are dedicated to respiration and that single Dbx1 preBötC neurons cannot fulfill multiple duties. Therefore, while their rhythmogenic role is well established at perinatal stages of development (Bouvier et al., 2010; Gray et al., 2010), the contemporary studies recapped above from adult mice indicate that rhythm generation may not be the principal function of Dbx1 preBötC neurons.

Here, we reevaluate the inspiratory rhythmogenic role of Dbx1 preBötC neurons in adult mice. We kept sensorimotor feedback intact because its removal otherwise slows the breathing rhythm and lowers preBötC excitability, which makes it more susceptible to perturbation. Thus, we test the role of Dbx1 neurons in the most realistic context *in vivo*. Using optogenetic technologies to photoinhibit or photostimulate Dbx1 neurons, we show that their perturbation affects breathing frequency and the precise timing of individual breaths within the breathing cycle, which are key properties of a core oscillator microcircuit. Other respiratory and non-respiratory roles notwithstanding, these data indicate that Dbx1 preBötC neurons constitute an essential core oscillator for inspiration.

## Materials and Methods

### Mice

The Institutional Animal Care and Use Committee at William & Mary approved these protocols, which conform to the policies of the Office of Laboratory Animal Welfare (National Institutes of Health) and the guidelines of the National Research Council of the US National Academy of Sciences.

Female mice that express tamoxifen-sensitive Cre recombinase in *Dbx1*-derived progenitor cells, i.e., *Dbx1* ^CreERT2^ (Ruangkittisakul et al., 2014; RRID:IMSR_JAX:028131) were mated with males from two different reporter strains. The first reporter strain expresses an archaerhodopsin-3 tagged with EGFP fusion protein (ArchT-EGFP) in a Cre-dependent manner (Allen Institute nomenclature, Ai40D; RRID:IMSR_JAX:021188). The second reporter strain features *Frt*- and *LoxP*-flanked STOP cassettes followed by a fusion gene coding for calcium translocating channelrhodopsin and EYFP (CatCh-EYFP), which is expressed following Cre- and Flp-mediated recombination (Allen Institute nomenclature, Ai80D; RRID:IMSR_JAX:025109). We administered tamoxifen to pregnant dams (22.5 mg/kg) at embryonic day 9.5 to maximize neuronal expression and minimize glial expression (Kottick et al., 2017). Dbx1;ArchT or Dbx1;CatCh mice were distinguished from wild-type littermates, which lack EGFP or EYFP, via *post hoc* histology. Therefore, wild-type littermates formed a control group whose constituent members were unknown to the experimenter.

### Brainstem slices

Neonatal Dbx1;ArchT mice (0–4 d old) were anesthetized via hypothermia, decerebrated, and then dissected in 4°C aCSF containing: 124 mM NaCl, 3 mM KCl, 1.5 mM CaCl_2_, 1 mM MgSO_4_, 25 mM NaHCO_3_, 0.5 mM NaH_2_PO_4_, and 30 mM dextrose aerated continually with carbogen (95% O_2_ and 5% CO_2_) at pH 7.4. The isolated neuraxes were glued to an agar block and mounted rostral side up in the vise of a vibratome. We cut the neuraxes in the transverse plane to obtain a single 500-µm-thick section containing the preBötC as well as the hypoglossal (XII) cranial motor nucleus and its rostral nerve rootlets. The anatomic criteria for isolating the preBötC in rhythmically active slices from neonatal Dbx1-reporter mice are detailed in a series of open access atlases (Ruangkittisakul et al., 2014). Slices were anchored using a silver wire grid in a recording chamber on a fixed-stage upright physiology microscope. We perfused slices with aCSF at 27° C (2 ml/min) and elevated the K^+^ concentration to 9 mM. Inspiratory motor output was recorded from the XII nerve rootlets using a differential amplifier (gain 2000×) and a bandpass filter (300–1000 Hz). Nerve root output was full-wave rectified and smoothed for display.

We identified Dbx1 neurons under epifluorescence via EGFP expression and then performed whole-cell patch-clamp recordings under visual control. Patch pipettes with tip resistance of 4–6 MΩ were fabricated from capillary glass (1.50 mm outer diameter, 0.86 mm inner diameter) and filled with solution containing: 140 mM potassium gluconate, 5 mM NaCl, 0.1 mM EGTA, 10 mM HEPES, 2 mM Mg-ATP, and 0.3 mM Na_3_-GTP. Alexa Fluor 568 hydrazide dye was added to the patch-pipette solution (50 µM, Invitrogen) as a color contrast to EGFP following whole-cell dialysis. Membrane potential was amplified (100×) and low-pass filtered (1 kHz) using a patch-clamp amplifier (EPC10, HEKA Elektronic) and digitally acquired at 4 kHz (PowerLab 4/30, AD Instruments).

### Virus injection and fiber optic implantation

We anesthetized adult Dbx1;ArchT and Dbx1;CatCh (aged 8–20 weeks) mice via intraperitoneal injection of ketamine (100 mg/kg) and xylazine (10 mg/kg) and performed aseptic surgeries in the prone position using a stereotaxic frame. After exposing the skull, we performed either one (Dbx1;CatCh mice) or two (Dbx1;ArchT mice) 0.5 mm diameter craniotomies in the range 6.95–7.07 mm posterior to bregma and 1.1–1.3 mm lateral to the midline suture.

In Dbx1;CatCh mice, we unilaterally injected an adeno-associated virus (AAV) immediately before fiber optic implantation to induce *Flp*-mediated recombination of *Frt* sites. We loaded an ultrafine, microvolume syringe (Neuros series, Hamilton) with 120 µl of AAV-eSyn-FLPo (titer 10^13^ vg/ml, catalog number VB1126, Vector Biolabs, RRID:SCR_011010). The syringe was lowered at 10 µm/s through the cerebellum and the virus was injected at the target site at ∼60 nl/min. The syringe remained in place for 10 min before being retracted at 10 µm/s.

Both Dbx1;ArchT and Dbx1;CatCh mice were equipped with fiber optic appliances constructed by joining 1.27-mm diameter ceramic ferrules (Precision Fiber Products) with 105-µm diameter 0.22 numerical aperture (NA) multimode fibers (Thorlabs). We implanted fiber optic appliances bilaterally in Dbx1;ArchT mice and unilaterally in Dbx1;CatCh mice at a depth of 5.5–5.9 mm from bregma, which were secured with a cyanoacrylate adhesive (Loctite 3092, Henkel Corp.). Dbx1;ArchT animals recovered for a minimum of 10 d before any further experimentation. Dbx1;CatCh mice recovered for a minimum of 21 d before further experimentation.

**Figure 1.**
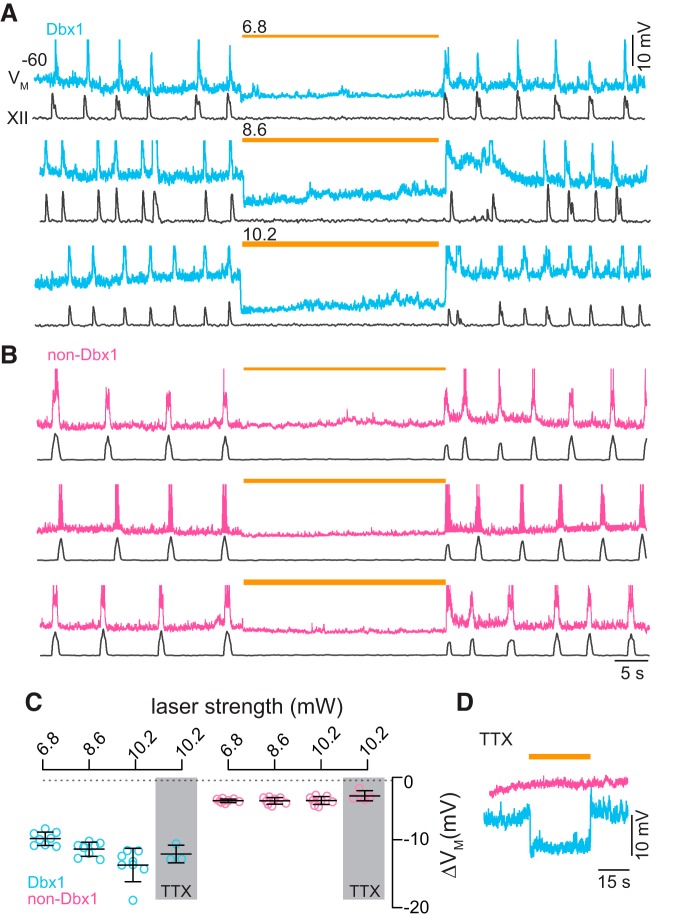
Photoinhibition of preBötC neurons *in vitro*. ***A***, Membrane trajectory of an ArchT-expressing Dbx1 preBötC neuron (V_M_, cyan traces) in a rhythmically active slice preparation from a neonatal Dbx1;ArchT mouse with inspiratory motor output recorded from the XII nerve rootlet (black traces). ***B***, Membrane trajectory of a non-Dbx1, non-ArchT-expressing preBötC neuron (V_M_, magenta traces) with XII motor output. Light pulses (30 s) were applied bilaterally to the preBötC at three intensities (units of mW) in ***A***, ***B***. Yellow line thickness corresponds to light intensity, which is also annotated above each line. Voltage and time calibrations apply to ***A***, ***B***, including baseline membrane potential of −60 mV. Action potentials have been truncated for display to emphasize the trajectory around the baseline membrane potential. ***C***, Membrane hyperpolarization (ΔV_M_) evoked by light pulses at three intensities in Dbx1 and non-Dbx1 preBötC neurons in aCSF and in the prsence of 1 µM TTX. Bars show mean and SD. ***D***, Membrane trajectories in response to 30-s bouts of 10.2-mW illumination in TTX.

We measured the membrane potential effects of ArchT activation in slices (Fig. 1). Because CatCh is not yet expressed at perinatal stages conducive to slice experiments, we were not able to measure CatCh effects on membrane potential directly. Using laser powers 6.8–10.2 mW (see below), we calculated the expected membrane depolarization according to measurements in Kleinlogel et al. (2011). Cultured hippocampal neurons virally transduced to express CatCh depolarized ∼50 mV in response to 473-nm light at 9.7 × 10^16^ photons/s·cm^2^. Laser power of 6.8–10.2 mW yields ∼1.6–2.5 × 10^16^ photons/s·cm^2^. Assuming that Dbx1 preBötC neurons *in vivo* respond similarly to hippocampal neurons in culture, the corresponding depolarization of Dbx1 preBötC neurons would be on the order of 8–15 mV. Two unknown factors may affect this estimate including how the presence of light-scattering white matter *in vivo* would attenuate light delivery, and how differences in input resistance between cultured hippocampal neurons and preBötC interneurons would impact CatCh-mediated currents’ ability to depolarize the different cell types.

### Breathing measurements

After anesthetizing mice using 2% isoflurane we connected the ferrules of Dbx1;ArchT mice to a 589-nm laser (Dragon Lasers). The ferrule of Dbx1;CatCh mice was connected to a 473-nm laser (Dragon Lasers). Mice recovered from isofluorane anesthesia for ∼1 h, and then, we measured breathing behavior using a whole-body plethysmograph (Emka Technologies) that allowed for fiber-optic illumination in a sealed chamber.

In a separate session, these same mice were lightly sedated via intraperitoneal ketamine injections (15 mg/kg minimum dose), which we titrated as needed to reduce limb movements but retain toe-pinch and blink reflexes. The maximum aggregate dose was limited to 50 mg/kg. Mice were fitted with a modified anesthesia mask (Kent Scientific) to measure breathing.

We applied a circuit of positive pressure, with balanced vacuum, to continuously flush the plethysmograph with breathing air. The plethysmograph and the mask were connected to a 1-l respiratory flow head and differential pressure transducer that measured airflow; positive airflow reflects inspiration in all cases. Analog breathing signals were digitized at 1 kHz (PowerLab).

### Optogenetic protocols

We applied 5-s bouts of light (either 473 or 589 nm) to Dbx1;ArchT and Dbx1;CatCh mice at graded intensities of 6.8, 8.6, and 10.2 mW. All ferrules were tested with a power meter before implantation to verify that illumination intensity did not vary >0.1 mW from the specified values. Bouts of light application were separated by a minimum interval of 30 s. Each mouse received a minimum of 10 presentations of each light stimulus (technical repeats). We also applied 100-ms light pulses at a fixed intensity of 10.2 mW. We exposed each mouse to 85–200 pulses spaced at random intervals of between 1 and 5 s.

We applied 589-nm light (at the same intensities listed above) to rhythmically active slices. The fiberoptics were targeted to selectively illuminate the preBötC bilaterally but not the adjacent reticular formation.

### Data analyses

The airflow signal was bandpass filtered (0.1–20 Hz) and analyzed using LabChart 8 software (AD Instruments), which computes airflow (units of ml/s), respiratory rate (i.e., frequency, ƒ, units of Hz), tidal volume (V_T_, units of ml), inspiratory time (T_i_, units of ms), and minute ventilation (MV; units of ml/min). We computed statistics using GraphPad Prism 6 and R: The Project for Statistical Computing (R, The R Foundation) and prepared figures using Adobe Illustrator (Adobe Systems Inc.), GraphPad Prism 6, and IGOR Pro 6 (Wavemetrics). We analyzed the experiments in which 5-s light pulses were applied to the preBötC using paired *t* tests, specifically comparing mean ƒ, V_T_, and MV for control and illumination conditions at three different light intensity levels (i.e., at each laser strength tested; the pre-illumination ventilation serves as its own control). At least five technical repeats were averaged to compute the mean ƒ, V_T_, and MV for each animal; the mean value for each animal represents one data point.

We analyzed phase-response relationships of the breathing cycles perturbed by 100 ms-duration light pulses. The expected cycle period was measured from the unperturbed cycle immediately before the light pulse, which was defined as spanning 0–360° (Φ_Expected_). Cycle times were measured from the start of inspiration in one breath to the start of inspiration of the subsequent breath. For perturbed cycles, 100-ms light pulses were applied at random time points spanning inspiration and expiration to test for phase shifts. Φ_Stim_ marks the phase at which the light pulse occurred. The induced cycle period (Φ_Induced_) was measured from the perturbed cycle. The perturbation of breathing phase, Φ_Shift_, was defined as the difference between Φ_Induced_ and Φ_Expected_. We calculated change in V_T_ and T_i_ in the perturbed breath compared to the expected breath normalized to the expected breath (referred to as, ΔV_T_ and ΔT_i_, respectively). Further, we calculated the phase shift of the breath following the perturbed breath (i.e., the cycle after Φ_Induced_) also with respect to Φ_Expected_; we refer to the phase of the subsequent breath Φ_N+1_. Measurements of Φ_Shift_, ΔV_T_, ΔT_i_, and Φ_N+1_ are all linked to a particular Φ_Stim_ within the interval 0-360°. To analyze group data, we sorted Φ_Stim_ into 12 equally sized 30° bins. We computed the mean and SD for Φ_Shift_, ΔV_T_, ΔT_i_, and Φ_N+1_ within each bin, which we then plotted in phase-response curves along with values calculated from wild-type littermates. A Tukey’s HSD test was used to evaluate how unlikely it would have been to obtain mean Φ_Shift_, ΔV_T_, ΔT_i_, and Φ_N+1_ for each bin if the optogenetic perturbations had commensurate effects on Dbx1;ArchT (or Dbx1;CatCh) mice and wild-type littermates.

### Histology

After experimentation we verified in all animals that fiber optic tips were within 500 µm of the dorsal preBötC border, which could be identified via well-established anatomic criteria in combination with either ArchT-EGFP or CatCh-EYFP fusion protein expression in reporter mice. We administered a lethal dose of pentobarbital (100 mg/kg, i.p.) and then transcardially perfused the mice with 1× PBS followed by 4% PFA in PBS. The neuraxes were removed and postfixed overnight in 4% PFA and later sliced in 50-µm contiguous transverse sections using a vibratome. Free-floating sections were stained using NeuroTrace 530/615 red fluorescent Nissl stain (Invitrogen) for 1 h, rinsed in PBS, and then cover-slipped using Vectashield (RRID:AB_2336789). Tissue sections were visualized using bright-field and confocal microscopy. Images were arranged as mosaics and brightness and contrast were adjusted uniformly across the entire ensemble image using the public domain software package ImageJ (RRID:SCR_003070). Images were not manipulated in any other way.

## Results

### ArchT activation hyperpolarizes Dbx1 preBötC neurons postsynaptically

We illuminated the preBötC in transverse medullary slices from neonatal Dbx1;ArchT mice that spontaneously generate inspiratory rhythm and airway-related hypoglossal (XII) motor output. Light application (589 nm) to the preBötC bilaterally stopped rhythm and motor output at all light intensities (Fig. 1*A*,*B*, black traces). Dbx1 preBötC neurons recorded in whole-cell patch-clamp hyperpolarized 6.5 ± 1.0, 8.1 ± 1.1, and 11.0 ± 2.5 mV in response to light of increasing intensity (Fig. 1*A*,*C*, cyan). We reapplied the highest intensity light in the presence of TTX, which hyperpolarized Dbx1 preBötC neurons by 8.6 ± 1.4 mV (Fig. 1*C*,*D*, cyan). Light-evoked hyperpolarization was commensurate before and after TTX (Mann–Whitney *U*, *p* = 0.3^a^), which suggests that ArchT hyperpolarizes Dbx1 preBötC neurons via direct postsynaptic effects.

In the same slices from neonatal Dbx1;ArchT mice, we illuminated the preBötC bilaterally while patch recording neighboring non-Dbx1 preBötC neurons. Baseline membrane potential in non-Dbx1 preBötC neurons responded negligibly to light, hyperpolarizing 0.7 ± 0.3, 1.1 ± 0.5, and 1.1 ± 0.6 mV in response to light of increasing intensity (Fig. 1*B*,*C*, magenta). In TTX, light at the highest intensity hyperpolarized non-Dbx1 neurons by 0.3 ± 0.8 mV (Fig. 1*C*, magenta), which was indistinguishable from light-evoked hyperpolarization before TTX application (Mann–Whitney *U*, *p* = 0.2^b^). These results suggest that light-evoked cessation of inspiratory rhythm and motor output *in vitro* is largely attributable to direct postsynaptic effects on Dbx1 preBötC neurons rather than network disfacilitation, which would comparably affect Dbx1 as well as non-Dbx1 neurons in the preBötC and would be eliminated by TTX.

### Photoinhibition of Dbx1 preBötC neurons attenuates breathing and resets inspiration

**Figure 2:**
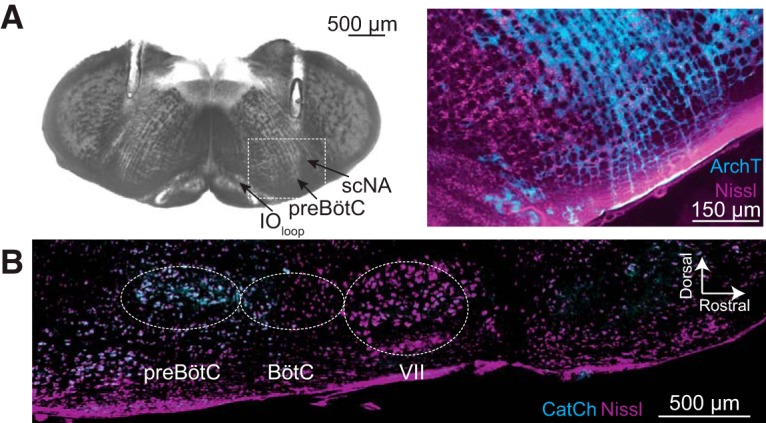
**. *A***, Bright field image of a transverse section from an adult Dbx1;ArchT mouse at the level the preBötC, as indicated by the loop of the inferior olive (IO_loop_) and the semi-compact division of the nucleus ambiguus (scNA). Parallel tracks of implanted fiber optics are visible from the dorsal border of the tissue section into the intermediate reticular formation dorsal to the preBötC. The selection box was imaged using fluorescence microscopy to show ArchT (cyan) protein expression in the preBötC in detail, Nissl staining (magenta) included for contrast. ***B***, Parasagittal section from an adult Dbx1;CatCh mouse. Nissl (magenta) shows anatomic landmarks including the facial (VII) cranial nucleus, Bötzinger complex (BötC), and the preBötC. CatCh (cyan) expression is limited to the preBötC.

**Figure 3.**
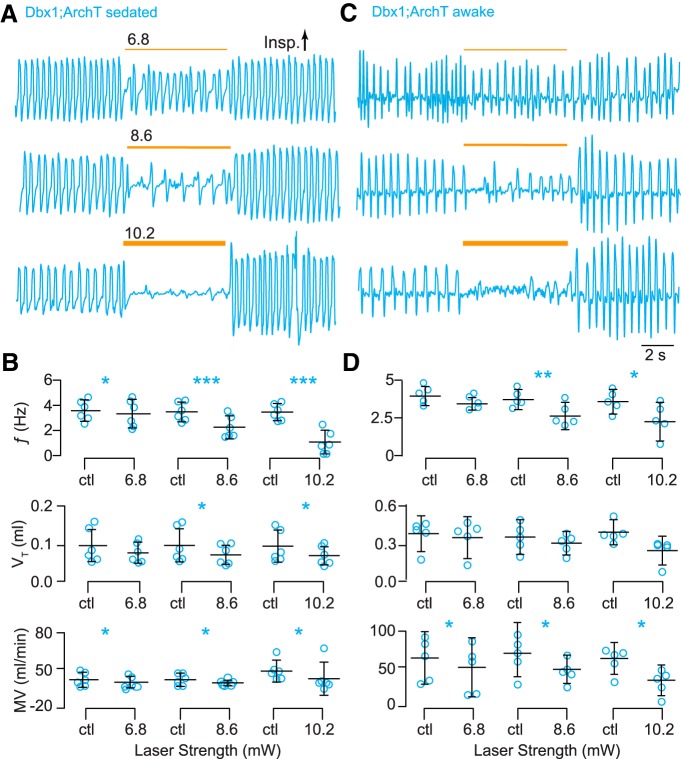
Photoinhibition of Dbx1 preBötC neurons depresses breathing in adult Dbx1;ArchT mice. ***A***, Airflow traces from a sedated mouse exposed to 5-s bouts of bilateral preBötC illumination at three intensities (units of mW). Yellow line thickness corresponds to light intensity, which is also annotated above each line. ***B***, Group data from experiments in A quantifying light-evoked changes in ƒ, V_T_, and MV. Symbols show the mean ƒ, V_T_, and MV measured in each mouse. Bars show the mean and SD for all animals tested (*n* = 6). Control measurements are labeled ctl; numerals indicate light intensity. ***C***, Airflow traces from an awake unrestrained mouse exposed to 5-s bouts of bilateral preBötC illumination at three intensities. Yellow line thickness corresponds to light intensity; annotations match those in ***A***. ***D***, Group data from experiments in ***C*** quantifying light-evoked changes in ƒ, V_T_, and MV. Symbols show the mean ƒ, V_T_, and MV measured in each mouse. Bars show the mean and SD for all animals tested (*n* = 5). Control measurements are labeled ctl; numerals indicate light intensity. Asterisks represent statistical significance at *p* < 0.05; the double asterisk represents *p* < 0.01; and triple asterisks represent *p* < 0.001.

Next, we illuminated the preBötC bilaterally using fiber-optic implants (Fig. 2*A* shows tracks of fiberoptics in *post hoc* histology) in sedated adult Dbx1;ArchT mice, which reduced breathing in all instances (Fig. 3*A*,*B*). In control conditions breathing frequency (ƒ) was typically ∼3.5 Hz, tidal volume (V_T_) was ∼0.1 ml, and MV was ∼50 ml/min. The lowest intensity light (6.8 mW) decreased ƒ by 0.3 Hz (*t* test, *p* = 0.0499^c^), did not change V_T_ (*t* test, *p* = 0.07^d^), and decreased MV by 9 ml/min (*t* test, *p* = 0.01^e^; Fig. 3*B*).

ƒ, V_T_, and MV decreased to a greater extent in response to 8.6 and 10.2 mW intensity illumination (Fig. 3*A*,*B*). ƒ decreased by 1.2 and 2.0 Hz, respectively (*t* test, *p* = 0.0006^f^ and *p* = 0.0003^g^). Apnea, no inspiratory effort, resulted in more than one-third of all trials at 10.2 mW (i.e., 11 of 30 bouts; Fig. 3*A*, bottom). V_T_ decreased in response to 8.6 and 10.2 mW light in both cases by 0.03 ml (*t* test, *p* = 0.04^h^ and *p* = 0.02^i^). MV decreased by 11 and 20 ml/min, respectively (*t* test, both *p* = 0.02^j^; Fig. 3*B*).

**Figure 4.**
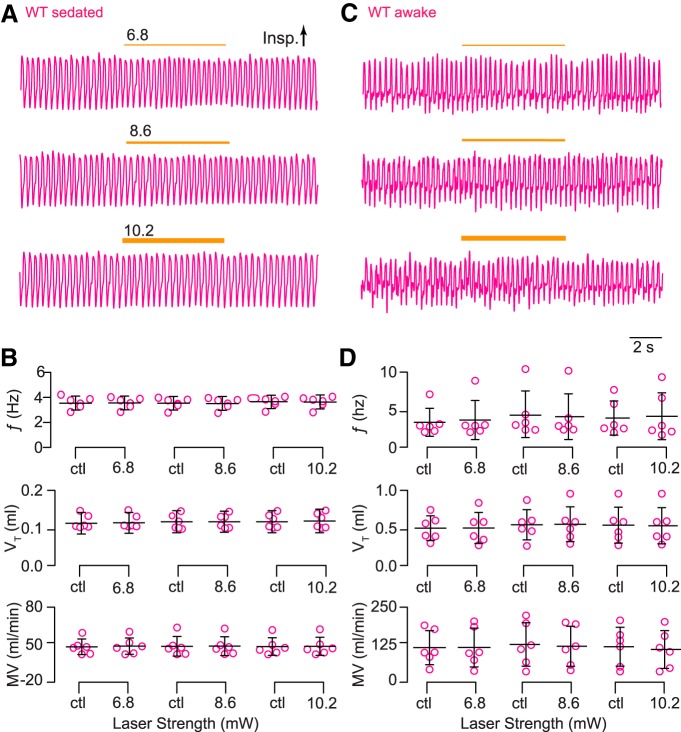
Light application to the preBötC does not affect breathing in wild-type Dbx1;ArchT littermates. ***A***, Airflow traces from a sedated mouse exposed to 5-s bouts of bilateral preBötC illumination at three intensities (units of mW). Yellow line thickness corresponds to light intensity, which is also annotated above each line. ***B***, Group data from experiments in A quantifying ƒ, V_T_, and MV in response to light application. Symbols show mean ƒ, V_T_, and MV in each mouse. Bars show the mean and SD for all animals tested (*n* = 6). Control measurements are labeled ctl; numerals indicate light intensity. ***C***, Airflow traces from an awake unrestrained mouse exposed to 5-s bouts of unilateral preBötC illumination at three intensities (units of mW). Yellow line thickness corresponds to light intensity; annotations match those in ***A***. ***D***, Group data from experiments in ***C*** quantifying ƒ, V_T_, and MV in response to light application. Symbols show mean ƒ, V_T_, and MV in each mouse. Bars show the mean and SD for all animals tested (*n* = 6). Control measurements are labeled ctl; numerals indicate light intensity.

In comparison, sedated wild-type littermates subjected to the same protocol showed no light-evoked changes in breathing (Fig. 4*A*,*B*).

We repeated these experiments in Dbx1;ArchT mice while awake and unrestrained (Fig. 3*C*,*D*). The lowest intensity light (6.8 mW) had no statistically significant effect on ƒ and V_T_ (*t* test, *p* = 0.06^k^ and 0.06^l^) but their product MV decreased significantly by 7.4 ml/min (*t* test, *p* = 0.04^m^; Fig. 3*D*).

The effects on breathing were more profound when we illuminated at 8.6 and 10.2 mW (Fig. 3*C*,*D*). ƒ decreased by 1.1 and 1.2 Hz, respectively (*t* test, *p* = 0.002^n^ and *p* = 0.02^°^) and MV decreased by 22 and 32 ml/min, respectively (*t* test, *p* = 0.02^p^ and *p* = 0.04^q^). One animal stopped breathing for ∼4 s (i.e., apnea; Fig. 3*C*, bottom trace). Statistical hypothesis testing did not detect significant light-induced changes in V_T_ (*t* test, *p* = 0.3^r^ and *p* = 0.09^s^), probably due to the high variability of V_T_ in awake animals (Fig. 3*D*).

In comparison, awake unrestrained wild-type littermates showed no changes in breathing in response to light of any intensity (Fig. 4*C*,*D*).

Therefore, these data collectively show that ArchT-mediated Dbx1 preBötC neuron hyperpolarization reduces breathing up to and including apnea in sedated and awake intact mice.

**Figure 5.**
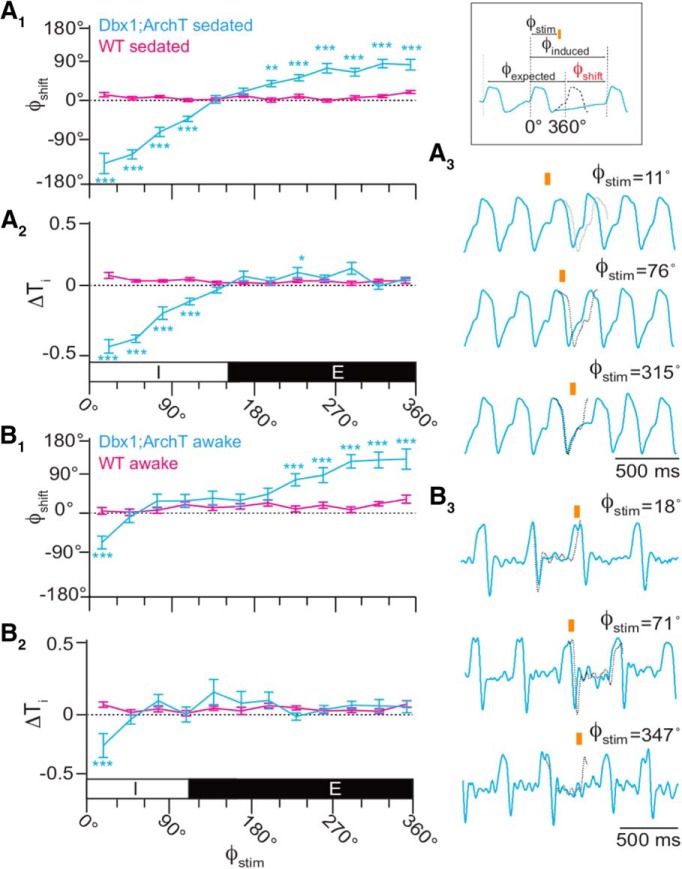
Effects of brief photoinhibition on the breathing phase and inspiratory duration in Dbx1;ArchT mice (*n* = 6 in ***A***, *n* = 5 in ***B***, cyan) and wild-type littermates (*n* = 6, magenta). ***A_1_***, Phase-response curve plotting Φ_Shift_ following 100-ms photoinhibition at Φ_Stim_ throughout the breathing cycle in sedated mice. Φ_Stim_ was partitioned into 12 equally sized bins (30°) in ***A***, ***B***. ***A_2_***, Phase-response curve showing changes in T_i_ following brief photoinhibition (i.e., the perturbed breath) in the same cohort of sedated mice. The abscissa marks the inspiratory (I, 0–150°) and expiratory (E, 150–360°) phases of the breathing cycle (0–360°), which applies to ***A_1_***, ***A_2_***. ***A_3_***, Sample airflow traces from a representative sedated mouse (Φ_Stim_ is indicated by an orange bar and numeral value). Time calibration is shown. Inset shows Φ_Stim_, Φ_expected_, Φ_induced_, and Φ_Shift_ as explained in the main text, where the change in phase is determined by the difference between the stimulated and induced phases. ***B_1_***, Phase-response curve plotting Φ_Shift_ following brief photoinhibition at Φ_Stim_ throughout the breathing cycle in awake unrestrained mice. ***B_2_***, Phase-response curve showing changes in T_i_ following brief photoinhibition (i.e., the perturbed breath) in the same cohort of awake unrestrained mice. The abscissa marks the inspiratory (I, 0–110°) and expiratory (E, 110–360°) phases of the breathing cycle (0–360°), which applies to ***B_1_***, ***B_2_***. ***B_3_***, Sample airflow traces from a representative awake unrestrained mouse (Φ_Stim_ is indicated by an orange bar and numeral value). Time calibration is shown.

Next, we applied brief (100 ms) light pulses randomly during the breathing cycle, which we defined as spanning 0–360° (see Materials and Methods; Fig. 5*A*, inset). Brief photoinhibition of the preBötC early during inspiration (Φ_Stim_ of 0–30°) caused a phase advance such that the subsequent inspiration occurred earlier than expected (Φ_Shift_ = −147 ± 23°, *p* = 1e-6^t^) while shortening inspiratory time (T_i_) by almost half (ΔT_i_ = 45 ± 5%, *p* = 1e-6^u^; Fig. 5*A_1_*,*A_2_*,*A_3_*, top trace). Brief photoinhibition also evoked significant phase advances and reduced T_i_ during the rest of inspiration (Φ_Stim_ of 30–120°), but the magnitude of those changes monotonically decreased as Φ_Stim_ approached the inspiratory-expiratory transition.

Brief photoinhibition did not perturb the system during the inspiratory-expiratory transition (Φ_Stim_ of 120–180°). During early expiration (Φ_Stim_ of 180–210°), which is often referred to as postinspiration (Dutschmann et al., 2014; Anderson et al., 2016), we observed the first significant phase delay such that the subsequent inspiration occurred later than expected in response to brief photoinhibition (Φ_Shift_ = 32 ± 7°, *p* = 0.006^v^; Fig. 5*A_1_*,*A_3_*, bottom trace). Phase delays were consistently evoked during expiration (Φ_Stim_ of 210–360°) with a maximum phase delay during late expiration (Φ_Stim_ of 300–330°; Φ_Shift_ = 78 ± 10°, *p* = 1e-6^w^). Brief photoinhibition during expiration did not affect T_i_, which is a straightforward result because the inspiratory period had ended (Fig. 5*A_2_*). Note, that ΔT_i_ was statistically significant at Φ_Stim_ of 210–240°) but that change is not physiologically meaningful because the magnitude of the change is small and not part of a consistent trend in the phase-response curve.

**Figure 6.**
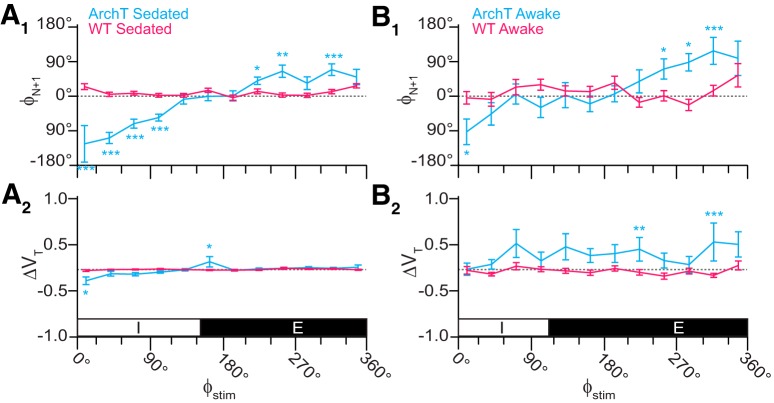
Effects of brief photoinhibition on V_T_ and Φ_N+1_ in Dbx1;ArchT mice (*n* = 5 in ***A***, *n* = 6 in ***B***, cyan) and wild-type littermates (*n* = 6, magenta). ***A_1_***, Phase-response curve plotting Φ_N+1_ versus Φ_Stim_ throughout the breathing cycle in sedated mice. ***A_2_***, Phase-response curve for changes in V_T_ following brief photoinhibition (i.e., the perturbed breath) in the same cohort of sedated mice. The abscissa marks the inspiratory (I, 0–150°) and expiratory (E, 150–360°) phases of the breathing cycle (0–360°), which applies to ***A_1_***, ***A_2_***. ***B_1_***, Phase-response curve plotting Φ_N+1_ versus Φ_Stim_ in awake unrestrained mice. ***B_2_***, Phase-response curve for ΔV_T_ versus Φ_Stim_ in the same cohort of awake unrestrained mice. The abscissa marks the inspiratory (I, 0–110°) and expiratory (E, 110–360°) phases of the complete breathing cycle (0–360°), which applies to ***B_1_***, ***B_2_***.

The relationship between Φ_Stim_ and the phase of the subsequent breath [Φ_N+1_ (Fig. 6*A_1_*) or Φ_N+2_ (data not shown)] closely resembled the relationship between Φ_Stim_ and Φ_Shift_ (Fig. 5*A_1_*), which suggests that brief photoinhibition resets the phase of the oscillator.

In contrast to its effects on breathing phase (Φ_Shift_ and Φ_N+1_), brief photoinhibition had little effect on V_T_ throughout most of the respiratory cycle with changes of <10% across the entire respiratory cycle, except during early inspiration (Φ_Stim_ of 0–30°, in which V_T_ decreased by 23 ± 8%, *p* = 0.002^x^) and early expiration (Φ_Stim_ of 150–180°, in which V_T_ increased by 16 ± 11%, *p* = 0.02^y^; Fig. 6*A_2_*). Despite the fact that two out of 12 measurements pass the threshold for statistical significance, these data do not convincingly demonstrate that brief photoinhibition of Dbx1 preBötC neurons systematically influences V_T_ in sedated mice.

We repeated brief photoinhibition experiments in awake unrestrained Dbx1;ArchT mice. The plots of Φ_Shift_, ΔT_i_, Φ_N+1_, and ΔV_T_ versus Φ_Stim_ were qualitatively similar to the experiments in sedated mice (Figs. 5 compare *A*, *B*, 6 compare *A*, *B*). Photoinhibition during early inspiration (Φ_Stim_ of 0–30°) caused a phase advance (Φ_Shift_ = −86 ± 16°, *p* = 1e-5^z^). The first significant phase delay in the awake animal occurred when brief photoinhibition was applied during peak expiration (Φ_Stim_ of 210–240°, Φ_Shift_ = 68 ± 15°, *p* = 1e-6^aa^). Φ_Shift_ tended to increase as brief photoinhibition was applied at later points during the expiratory phase. The maximum phase delay occurred during late expiration (Φ_Stim_ of 330–360°, Φ_Shift_ = 118 ± 25°, *p* = 4e-5^bb^; Fig. 5*B_1_*,*B_3_*). Brief photoinhibition decreased T_i_ by nearly one-third (ΔT_i_ = 28 ± 9%, *p* = 1e-5^cc^) during early inspiration (Φ_Stim_ of 0–30°) but had no significant effect at any other time during the cycle.

### Photostimulation of Dbx1 preBötC neurons enhances breathing and modifies the timing and magnitude of breaths

**Figure 7.**
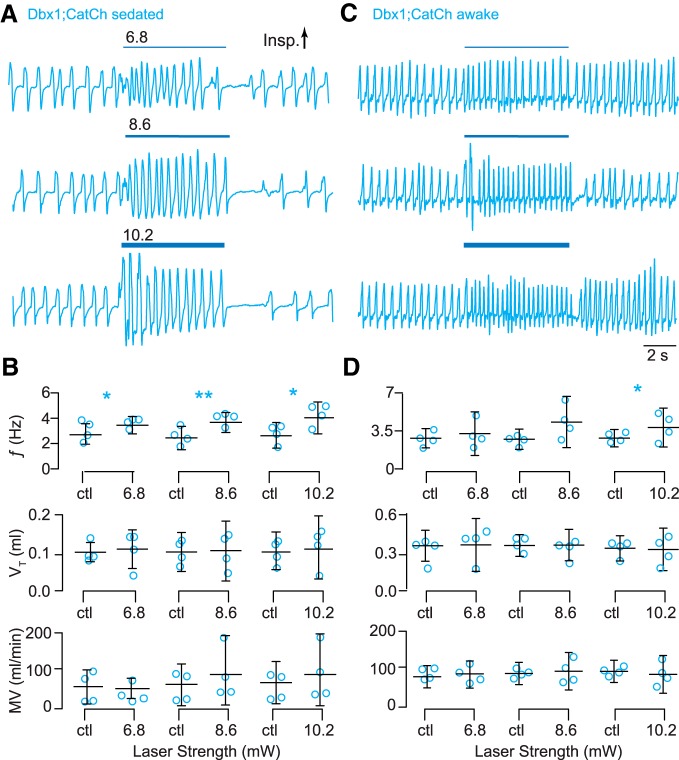
Photostimulation of Dbx1 preBötC neurons speeds-up breathing in adult Dbx1;CatCh mice. ***A***, Airflow traces from a sedated mouse exposed to 5-s bouts of unilateral preBötC illumination at three intensities (units of mW). Cyan line thickness corresponds to light intensity, which is also annotated above each line. ***B***, Group data from experiments in ***A*** quantifying light-evoked changes in ƒ, V_T_, and MV. Symbols show the mean ƒ, V_T_, and MV measured in each mouse. Bars show the mean and SD for all animals tested (*n* = 4). Control measurements are labeled ctl; numerals indicate light intensity. ***C***, Airflow traces from an awake unrestrained mouse exposed to 5-s bouts of bilateral preBötC illumination at three intensities. Cyan line thickness corresponds to light intensity; annotations match those in ***A***. ***D***, Group data from experiments in ***C*** quantifying light-evoked changes in ƒ, V_T_, and MV. Symbols show the mean ƒ, V_T_, and MV measured in each mouse. Bars show the mean and SD for all animals tested (*n* = 4). Control measurements are labeled ctl; numerals indicate light intensity. Asterisks represent statistical significance at *p* < 0.05; the double asterisk represents *p* < 0.01.

We illuminated the preBötC unilaterally in sedated adult Dbx1;CatCh mice following viral transduction in the preBötC with a synapsin-driven Flp recombinase. This double-stop intersectional approach limited CatCh-EYFP expression to the preBötC (Fig. 2*B*). In control conditions ƒ was typically ∼3 Hz, V_T_ was ∼0.1 ml, and MV was ∼50 ml/min. Bouts of blue light (473 nm) at three intensities significantly increased ƒ by 0.8, 1.1, and 1.3 Hz, respectively (*t* test, *p* = 0.03^dd^, 0.005^ee^, and 0.03^ff^). There were no significant effects on V_T_ or MV at any light intensity (Fig. 7*A*,*B*).

We repeated these unilateral photostimulation experiments in Dbx1;CatCh mice while awake and unrestrained. Frequency increased by 1.6 Hz in response to light at the highest intensity (*t* test, *p* = 0.04^gg^; Fig. 7*C*,*D*). There were no other notable changes in ƒ, V_T_, or MV at any light intensity.

**Figure 8.**
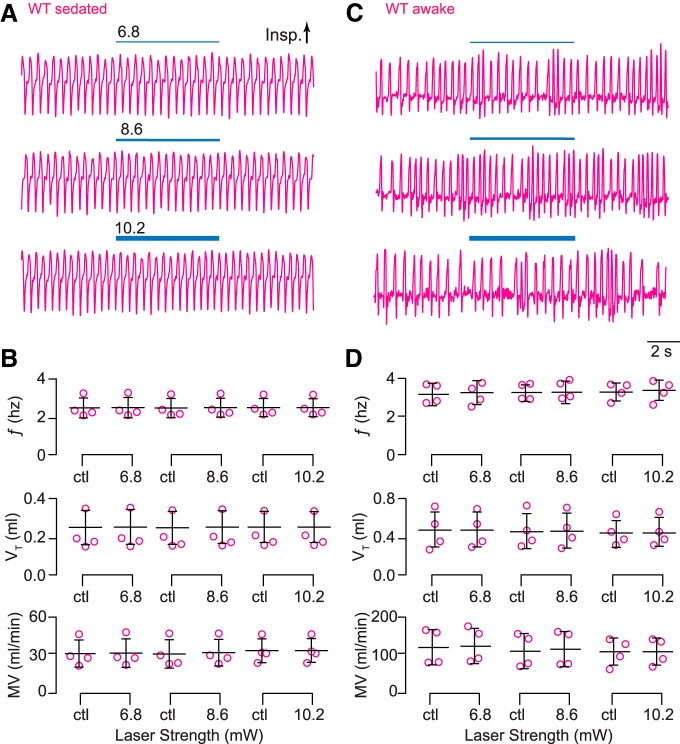
Light application to the preBötC does not affect breathing in wild-type Dbx1;CatCh littermates. ***A***, Airflow traces from a sedated mouse exposed to 5-s bouts of unilateral preBötC illumination at three intensities (units of mW). Cyan line thickness corresponds to light intensity, which is also annotated above each line. ***B***, Group data from experiments in A quantifying ƒ, V_T_, and MV in response to light application. Symbols show mean ƒ, V_T_, and MV in each mouse. Bars show the mean and SD for all animals tested (*n* = 4). Control measurements are labeled ctl. ***C***, Traces from an awake unrestrained mouse exposed to 5-s bouts of unilateral preBötC illumination at three intensities. Cyan line thickness corresponds to light intensity; annotations match those in ***A***. ***D***, Group data from experiments in ***C*** quantifying ƒ, V_T_, and MV in response to light application. Symbols show mean ƒ, V_T_, and MV in each mouse. Bars show the mean and SD for all animals tested (*n* = 4). Control measurements are labeled ctl; numerals indicate light intensity.

In wild-type littermates, we observed no effects on breathing in either sedated or awake mice in response to light at any intensity (Fig. 8).

Therefore, these data collectively show that CatCh-mediated photostimulation of Dbx1 preBötC neurons selectively enhances breathing frequency in sedated and, to a limited extent, awake mice.

**Figure 9.**
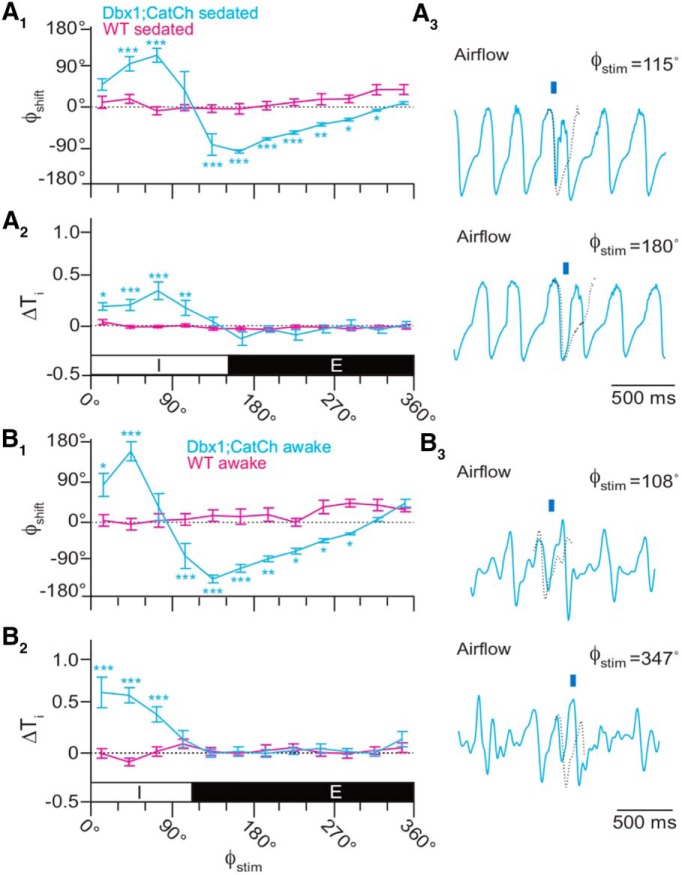
Effects of brief photostimulation on the breathing phase and inspiratory duration from Dbx1;CatCh mice (*n* = 4, cyan) and wild-type littermates (*n* = 4, magenta). ***A_1_***, Phase-response curve plotting Φ_Shift_ following 100-ms photostimulation at Φ_Stim_ throughout the breathing cycle in sedated mice. Φ_Stim_ was partitioned into 12 equally sized bins (30°) in ***A***, ***B***. ***A_2_***, Phase-response curve for changes in T_i_ following photostimulation (i.e., the perturbed breath) in the same cohort of sedated mice. The abscissa marks the inspiratory (I, 0–150°) and expiratory (E, 150–360°) phases of the breathing cycle (0–360°), which applies to ***A_1_***, ***A_2_***. ***A_3_***, Sample airflow traces from a representative sedated mouse (Φ_Stim_ is indicated by an orange bar and numeral value). Time calibration as shown. ***B_1_***, Phase-response curve plotting Φ_Shift_ following brief photostimulation at Φ_Stim_ throughout the breathing cycle in awake unrestrained mice. ***B_2_***, Phase-response curve for changes in T_i_ following brief photostimulation (i.e., the perturbed breath) in the same cohort of awake unrestrained mice. The abscissa marks the inspiratory (I, 0–110°) and expiratory (E, 110–360°) phases of the complete breathing cycle (0–360°), which applies to ***B_1_***, ***B_2_***. ***B_3_***, Sample airflow traces from a representative awake unrestrained mouse (Φ_Stim_ is indicated by an orange bar and numeral value). Time calibration is shown.

**Figure 10.**
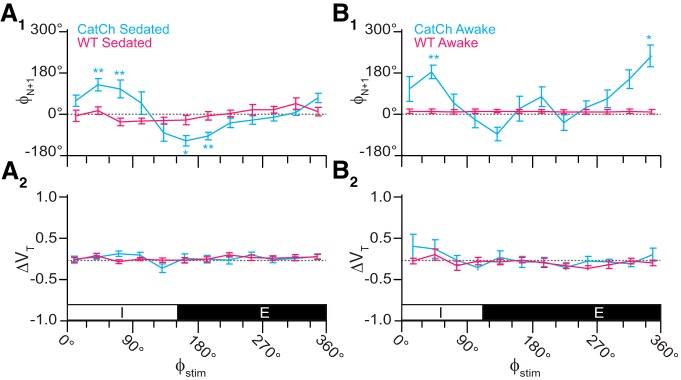
Effects of brief photostimulation on V_T_ and Φ_N+1_ in Dbx1;CatCh mice (*n* = 4, cyan) or wild-type littermates (*n* = 4, magenta). ***A_1_***, Phase-response curve plotting Φ_N+1_ versus Φ_Stim_ throughout the breathing cycle in sedated mice. ***A_2_***, Phase-response curve for changes in V_T_ following photostimulation (i.e., the perturbed breath) in the same cohort of sedated mice (*n* = 4). The abscissa marks the inspiratory (I, 0–150°) and expiratory (E, 150–360°) phases of the breathing cycle (0–360°), which applies to ***A_1_***, ***A_2_***. ***B_1_***, Phase-response curve plotting Φ_N+1_ versus Φ_Stim_ in awake unrestrained mice. ***B_2_***, Phase-response curve for ΔV_T_ versus Φ_Stim_ in the same cohort of awake unrestrained mice. The abscissa marks the inspiratory (I, 0–110°) and expiratory (E, 110–360°) phases of the complete breathing cycle (0–360°), which applies to ***B_1_***, ***B_2_***.

Next, we applied brief (100 ms) light pulses at different time points during the breathing cycle. Unilateral illumination of the preBötC during inspiration caused a phase delay and increased T_i_. The maximum phase delay occurred during peak inspiration (Φ_Stim_ of 60–90°, Φ_Shift_ = 125 ± 18°, *p* = 1e-6^hh^; Fig. 9*A_1_*) and coincided with the maximum ΔT_i_ (29 ± 7%, *p* = 1e-6^ii^; Fig. 9*A_2_*). Brief photostimulation caused a phase advance during the inspiratory-expiratory transition (Φ_Stim_ of 120–150°) and throughout expiration (Φ_Stim_ ≥ 150°) without affecting T_i_ (Fig. 9*A_1_*,*A_2_*,*A_3_*). The maximum phase advance occurred during early expiration (Φ_Stim_ of 150–180°, Φ_Shift_ = −128 ± 4°, *p* = 1e-6^jj^). The relationship between Φ_Stim_ and the phase of the subsequent breath [Φ_N+1_ (Fig. 10*A_1_*) or Φ_N+2_ (data not shown)] mimicked the relationship between Φ_Stim_ and Φ_Shift_ (Fig. 9*A_1_*), which suggests that brief photostimulation resets the phase of the oscillator. We observed no effects of brief photostimulation on V_T_ (Fig. 10*A_2_*).

We repeated brief photostimulation experiments in awake intact Dbx1;CatCh mice. The plots of Φ_Shift_ and ΔT_i_ versus Φ_Stim_ were qualitatively similar to those recorded in sedated mice (Figs. 9 compare *A*, *B*, 10 compare *A*, *B*). Brief photostimulation during early and mid-inspiration (Φ_Stim_ of 0–60°) caused a phase delay (maximum Φ_Shift_ = 147 ± 52, *p* = 1e-5^kk^; Fig. 9*B_1_*). We measured no phase shift for late inspiration (Φ_Stim_ of 60–90°). The phasic effect of brief photostimulation changed sign around the inspiratory-expiratory transition (Φ_Stim_ ≥ 90°); brief photostimulation subsequently evoked breaths earlier than expected. We measured the maximum phase advance during early expiration (Φ_Stim_ of 120–150°, Φ_Shift_ = −159 ± 9°, *p* = 1e-5^ll^; Fig. 9*B_1_*). The last statistically significant phase delay occurred during late expiration (Φ_Stim_ of 270–300°, Φ_Shift_ = −52 ± 3°, *p* = 0.0499^mm^).

Brief photostimulation of Dbx1 preBötC neurons in awake intact mice also extended T_i_ during inspiration (Fig. 9*B_2_*); the effect was even more pronounced than in sedated mice (Fig. 9*A_2_*). The maximum ΔT_i_ occurred during early inspiration (Φ_Stim_ of 0–30°) in which T_i_ increased by over half (56 ± 14%, *p* = 1e-6^nn^). The ability of brief photostimulation to extend T_i_ decreased during the inspiratory phase (Fig. 9*B_2_*) such that no significant effects occurred after Φ_Stim_ exceeded 90°. The relationship between Φ_Stim_ and Φ_N+1_ (or Φ_N+2_; data not shown) illustrated a phase delay evoked by brief photostimulation during mid-inspiration (Φ_Stim_ of 30–60°; Fig. 10*B_1_*), which partially recaps the relationship that was more pronounced in the plot of Φ_Shift_ versus Φ_Stim_ (Fig. 9*B_1_*). We observed no relationship for ΔV_T_ versus Φ_Stim_ (Fig. 10*B2*), as in the sedated mouse (Fig. 10*A_2_*).

These data are consistent photostimulus-induced resetting of the inspiratory oscillator, although the data are noisier in the awake adult, freely behaving mouse.

## Discussion

### Role diversity challenges the Dbx1 core hypothesis

The idea that Dbx1 preBötC neurons are inspiratory rhythmogenic has become generally well accepted, but it must be reevaluated given the expanding spectrum of non-rhythmogenic and non-respiratory functions attributed to this neuron class, particularly in adult animals.

Perinatally Dbx1 preBötC neurons generate rhythm and pattern. *Dbx1* knock-out mice do not breathe and form no recognizable preBötC (Bouvier et al., 2010; Gray et al., 2010), the site of inspiratory rhythmogenesis (Smith et al., 1991; Feldman and Del Negro, 2006; Feldman et al., 2013; Ramirez et al., 2016; Del Negro et al., 2018). Their selective destruction in a slice model of breathing (Funk and Greer, 2013) slows and then stops the rhythm, evidence of their rhythmogenic role, while also attenuating airway-related XII motor output (Wang et al., 2014) because of Dbx1 premotor neurons in the preBötC that drive XII (Wang et al., 2014; Revill et al., 2015) as well as phrenic motoneurons (Wu et al., 2017).

This theme continues in adult mice. Sst-expressing preBötC neurons, ∼17% of the Dbx1-derived population, appear to lack rhythmogenic function but rather shape motor output pattern (Cui et al., 2016; but see Koizumi et al., 2016). More than half (56%) of Dbx1 preBötC neurons characterized by Cdh9 expression lack respiratory rhythmicity but project to the locus coeruleus and putatively influence arousal (Yackle et al., 2017). If we assume that non-Sst and non-Cdh9 Dbx1 neurons have respiratory functions, and that individual neurons do not fulfill multiple duties, then these statistics suggest that not >27% of Dbx1 preBötC neurons in adult mice are exclusively rhythmogenic.

### Photoinhibition and photostimulation demonstrate Dbx1 preBötC neurons influence rhythm and pattern

Sustained photoinhibition caused graded frequency decreases including apnea, which are evidence that Dbx1 neurons form the core oscillator. However, photoinhibition also decreased V_T_, indicating that Dbx1 neurons also govern breath size, i.e., motor pattern. We previously reported qualitatively similar data (Vann et al., 2016), but the effects were milder because of the weaker archaerhodopsin variant available at the time. Dbx1 neurons that influence airway muscles and the diaphragm have been analyzed in detail (Wang et al., 2014; Revill et al., 2015; Cui et al., 2016; Wu et al., 2017). Here, we limit our comments to acknowledging those motor-related roles, and we concentrate on analyzing the role of Dbx1 preBötC neurons in rhythmogenesis.

Sustained photostimulation approximately doubled the breathing rate from ∼3.5–7 Hz. In contrast, Baertsch et al. (2018) reported minor (∼10%) frequency changes in vagus intact mice in response to sustained photostimulation. These two results are not discrepant, even if they appear to be at face value. We were able to evoke higher frequencies in our experiments most likely due to the accelerated response time, enhanced light sensitivity, larger voltage responses evoked by photoactivated CatCh compared to ChR2 (Kleinlogel et al., 2011), and the fact that we applied laser strengths up to 10.2 mW, whereas Baertsch et al. (2018) purposely limited their pulses to 7 mW or less. Those authors showed that phasic synaptic inhibition critically influences breathing frequency and we do not disagree. We purposely did not vagotomize our mice to preserve phasic synaptic inhibition and thus high breathing frequencies are possible during photostimulation.

### Phase-response experiments demonstrate that Dbx1 preBötC neurons are rhythmogenic

If Dbx1 preBötC neurons are inspiratory rhythmogenic, then transiently stimulating them should evoke inspiratory breaths at any point in the breathing cycle except, potentially, during the postinspiratory (early expiratory) refractory period identified *in vitro* (Guerrier et al., 2015; Kottick and Del Negro, 2015) and in vagotomized mice *in vivo* (Baertsch et al., 2018). We evoked inspiratory breaths at all points during the respiratory cycle without evidence of a refractory period. Brief photostimulation during inspiration prolonged it (i.e., increased T_i_) and delayed the next cycle (i.e., a phase delay). The straightforward interpretation is that CatCh-mediated inward current augments recurrent excitation thus prolonging inspiratory burst duration. Overexcited rhythmogenic neurons require more time to recover, which lengthens cycle time and delays the subsequent inspiration.

We observed that photostimulation at any other point in the cycle evoked inspiration earlier than expected, a phase advance, but did not otherwise modify inspiration. Our present results contrast a prior report in which brief photostimulation did not evoke phase advances during early expiration (Alsahafi et al., 2015). But in that experimental context, a synapsin promoter drove channelrhodopsin expression in both excitatory and inhibitory preBötC neurons. Because preBötC rhythmogenesis depends on recurrent excitation, and the network is at the nadir of its excitability during early expiration (Feldman and Kam, 2015; Ramirez et al., 2016; Del Negro et al., 2018), photostimulation of inhibitory neurons in concert with excitatory neurons would be less effective to evoke inspiratory bursts during early expiration.

Selective photostimulation of excitatory Dbx1-derived preBötC neurons should evoke phase advances during early expiration, and it does. Cui et al. (2016) photostimulated excitatory Dbx1 neurons and evoked phase advances of up to ∼72° during most of expiratory phase, except during the inspiratory-expiratory transition. We evoked more substantial phase advances of 90–150° during the early expiration. These results are not in conflict, but key methodological differences may explain the discrepancy. Cui et al., anesthetized their mice and applied a maximum laser power of 7 mW to activate channelrhodopsin, whereas we used awake or lightly sedated mice and applied a maximum laser power of 10.2 mW to activate the channelrhodopsin variant CatCh. Assuming that the fiber-optic appliances in both studies equally attenuate laser power from box to preBötC, then the larger phase advances we evoked during early expiration could be attributable to a higher excitability level of the preBötC in the unanesthetized (or lightly sedated) mice, higher laser power, as well as the accelerated response time, enhanced light sensitivity, and larger voltage responses evoked by photoactivated CatCh compared to ChR2 (Kleinlogel et al., 2011).

Brief photoinhibition of Dbx1 preBötC neurons during inspiration shortened it (i.e., decreased T_i_) and initiated the next cycle earlier than expected, a phase advance. We infer that hyperpolarizing rhythmogenic neurons checks the recurrent excitation process, which impedes but does not prevent inspiration. Nevertheless, the evoked breath is shorter in duration. preBötC neurons do not overexcite or become refractory, which facilitates the onset of the next cycle, hence the phase advance. That mechanism, here evoked by ArchT, mirrors the role of endogenous phasic synaptic inhibition, which curbs recurrent excitation to limit inspiratory activity and facilitate inspiratory-expiratory phase transition (Baertsch et al., 2018). We found that photoinhibition during expiration consistently caused a phase delay, which indicates hyperpolarization of Dbx1 preBötC neurons impedes recurrent excitation and thus prolongs the interval until the next inspiration.

The phase advances and phase delays induced by transient photoinhibition and photostimulation were recapitulated in phase plots of subsequent cycles (Φ_N+1_, Φ_N+2_). Those data indicate that transient optogenetic perturbation acts on, and resets, the phase of the core oscillator.

Our interpretations of the phase-response and resetting experiments, both photostimulation and photoinhibition, are consistent with Dbx1 preBötC neurons having direct temporal control over inspiration as well as postinspiration and the expiratory interval. That conclusion may seem overly broad considering, first, that the preBötC is the acknowledged inspiratory oscillator and, second, that oscillator microcircuits for postinspiration (Anderson et al., 2016) and expiration (Pagliardini et al., 2011; Huckstepp et al., 2015, 2016) also exist. Nevertheless, the preBötC plays a dominant role in organizing all phases of breathing by entraining the other oscillators in intact mice, and in reduced preparations that retain PiCo and pF_L_ (Moore et al., 2013; Ramirez et al., 2016; Del Negro et al., 2018). Therefore, the present data are consistent with Dbx1 preBötC interneurons constituting the oscillator core for inspiration and the central organizer for breathing.

### Could optogenetic perturbation of inputs to the preBötC modulate breathing?

The intersectional mouse genetics in Dbx1;ArchT mice leads to fusion protein expression in Dbx1-derived cells throughout the neuraxis. Therefore, preBötC illumination inhibits constituent interneurons but also axons of passage and the axon terminals of Dbx1 neurons from remote locations (Ruangkittisakul et al., 2014) that could disfacilitate the preBötC. If disfacilitation were primarily modulating preBötC activity in Dbx1;ArchT mice, then light-evoked hyperpolarization should be commensurate in non-Dbx1 neurons (which do not express ArchT) and Dbx1 neurons; and, TTX should block it in both cases. However, non-Dbx1 neurons hyperpolarized ∼1 mV in response to maximum illumination whereas Dbx1 neurons hyperpolarized ∼11 mV, and TTX did not notably affect either response. We conclude that direct postsynaptic hyperpolarization of Dbx1 preBötC neurons, rather than a reduction of tonic excitatory drive, is the predominant effect of preBötC illumination in Dbx1;ArchT mice.

Light-evoked breathing changes in Dbx1;CatCh mice cannot be explained by photostimulation of axon terminals and axons of passage that originate outside of, but synapse within, the preBötC. We used double-stop technology to limit CatCh expression to Dbx1-derived neurons (not glia, see below), whose somas reside in the preBötC or directly adjacent sites including the Bötzinger complex of inhibitory neurons (Ezure et al., 2003; Tanaka et al., 2003), and the rostral ventral respiratory group (Ellenberger and Feldman, 1990; Dobbins and Feldman, 1994; Gaytán et al., 2002) of excitatory phrenic premotor neurons. If Dbx1-derived expiratory neurons in the Bötzinger complex exist (which has not been demonstrated), then their photostimulation would depress breathing (Janczewski et al., 2013; Marchenko et al., 2016), the opposite of what we measured. If photostimulation affected Dbx1 phrenic premotor neurons in the rostral ventral respiratory group (Wu et al., 2017), then that would enhance the magnitude of inspiratory breaths, but not the inspiratory timing circuits in the preBötC. Sustained photostimulation experiments only enhanced breathing frequency and never V_T_, which diminishes the likelihood that our protocols influenced Dbx1-derived phrenic premotoneurons. Therefore, this caveat, the potential expression of CatCh in regions bordering the preBötC, is unlikely to affect our primary conclusions regarding rhythmogenesis.

### Effects on Dbx1-derived glia in the preBötC

Dbx1-expressing precursor cells develop into neurons and glia (Bouvier et al., 2010; Gray et al., 2010; Ruangkittisakul et al., 2014; Kottick et al., 2017), but optogenetic perturbation of glia is unlikely to have influenced the present results. First consider photoinhibition. Astrocytes support excitatory synaptic function in the preBötC (Hülsmann et al., 2000), but that role is metabolic in nature and light-evoked hyperpolarization would not preclude it. Calcium excitability and gliotransmission, which could be affected by photoinhibition, pertain to purinergic modulation and hypoxic challenges to the preBötC (Huxtable et al., 2010; Angelova et al., 2015; Funk et al., 2015; Rajani et al., 2017) but are less relevant factors governing the basal breathing state, which is the baseline for our experiments.

Photostimulation experiments unambiguously identify Dbx1 neurons (not glia) as the cellular population that forms the core inspiratory oscillator. CatCh expression was induced following Cre/Lox and Frt/Flp recombination. We used a synapsin promoter to express Flp locally in the preBötC so only Dbx1 neurons would be transduced and express CatCh.

ArchT expression is selectively (but not exclusively) limited to neurons by the timing of tamoxifen administration. Inducing Cre/lox recombination in pregnant *Dbx1* ^CreERT2^ mice at E9.5 reduces ArchT expression in glia to ∼40%, whereas ArchT expression in neurons remains above 90% (Kottick et al., 2017), which increases our confidence that photoinhibition largely affects neurons (not glia) and that neurons are the predominate rhythmogenic constituents and most parsimonious explanation for the light-induced changes in breathing.

Nevertheless, we are left with this disparity: ArchT activation is able to suppress breathing frequency more than CatCh activation is able to augment it. From baseline breathing rates *in vivo*, photoinhibition of Dbx1 excitatory neurons appears to have a more profound effect on frequency by slowing the recurrent excitation process, although we cannot negate that ArchT-mediated photoinhibition of Dbx1-derived glia removes gliotransmitter (perhaps purinergic) drive to the preBötC rhythmogenic network as well, which also diminishes frequency. In contrast, CatCh-mediated depolarization of Dbx1 neurons probably has a less profound frequency effect because elevating breathing rate above basal rates *in vivo* depends to a far greater extent on phasic synaptic inhibition rather than excitatory drive (Cregg et al., 2017; Baertsch et al., 2018), although the lack of photostimulation of gliotransmission could contribute to the diminished frequency effect too.

### Size of the Dbx1 core oscillator

Up to 73% of Dbx1 preBötC neurons serve non-rhythmogenic functions: 56% influence arousal (Yackle et al., 2017) and 17% influence motor pattern (Cui et al., 2016), which accounts nearly three-quarters of the Dbx1 population in the preBötC. What implications does that have for the composition and size of the inspiratory core oscillator whose constituent interneurons are Dbx1-derived too?

Dbx1-Cdh9 preBötC neurons were certainly photoinhibited and photostimulated in our experiments. However, those neurons influence behavioral state (e.g., eupnea, grooming, exploring, sniffing, etc.) rather than cycle-to-cycle breathing dynamics. We applied optogenetic perturbations only during eupnea, not during grooming or active movement, to control for behavioral shifts. Given that Dbx1-Cdh9 neurons are either weakly or not rhythmic (Yackle et al., 2017), briefly perturbing them would not influence the phase-response relationships, and thus would not confound our interpretation that Dbx1 preBötC neurons (even if a limited fraction of them) comprise the core oscillator.

Illumination of Sst-expressing Dbx1 neurons could be responsible for the decreases in V_T_ and apneas we report during sustained photoinhibition. In general, perturbations of Sst-expressing preBötC neurons affect breathing motor pattern in vagotomized and non-vagotomized adult mice and reduced *in situ* preparations (Cui et al., 2016; Koizumi et al., 2016); the depression of Sst-expressing preBötC neurons is strong enough to completely stop breathing movements in intact adult rats (Tan et al., 2008). Our experiments would only impact neurons that are both Dbx1-dervied and Sst-expressing, thus a smaller population than Tan et al. (2008) manipulated. Nevertheless, to the extent that photoinhibition decreased breath magntidue and caused apnea, we attribute that in part to direct effects on pattern-related Sst-expressing Dbx1-derived preBötC neurons that are either premotor part of a larger pattern-generating system (Revill et al., 2015; Cui et al., 2016; Wu et al., 2017).

If Cdh9 and Sst subpopulations of Dbx1 preBötC neurons are independent of the core respiratory oscillator, then only a small fraction (∼27%) of Dbx1 neurons are available for rhythmogenesis. Dbx1 neurons that comprise the preBötC core number ∼600 (Wang et al., 2014; Kottick et al., 2017). If one excludes Cdh9 and Sst neurons from this estimation, then as few as 160 Dbx1 preBötC neurons would remain for rhythmogenesis (we assume subpopulations serve one function). Can such a small number of interneurons comprise the inspiratory core oscillator?

Holographic photolysis of caged glutamate onto four to nine preBötC neurons evokes inspiratory motor output *in vitro* (Kam et al., 2013). This type of stimulation would affect Dbx1-Cdh9 neurons that are weakly or non-rhythmic (Kam et al., 2013; Yackle et al., 2017) as well as inhibitory preBötC neurons (Kuwana et al., 2006; Winter et al., 2009; Morgado-Valle et al., 2010) so it may overestimate the minimum number of activated preBötC neurons needed to evoke inspiratory bursts. Regardless, a reasonable conclusion is that stimulating relatively small numbers of preBötC neurons are capable of inducing inspiratory burst cycles, which lends credence to the notion that a small subfraction of Dbx1 preBötC neurons could be rhythmogenic in the midst of a potentially larger population of non-rhythmogenic (both pattern-generating and non-respiratory) preBötC neurons.

Glutamatergic preBötC neurons not derived from Dbx1-expressing precursors may also comprise part of the core oscillator (Koizumi et al., 2016; Baertsch et al., 2018). We cannot precisely estimate the size of that subpopulation but we expect that it will be small based on the small fraction of preBötC neurons that express Vglut2 but not Dbx1 (Bouvier et al., 2010; Gray et al., 2010).

### Dbx1 core hypothesis

The rhythmogenic subset of Dbx1 preBötC interneurons may be small, perhaps as little as 27% of the total Dbx1 population, but its outsize contribution to rhythmogenesis is unmistakable given the robust effects of sustained and transient photoinhibition and photostimulation demonstrated here, and by prior reports (Alsahafi et al., 2015; Cui et al., 2016; Koizumi et al., 2016). Therefore, whatever else Dbx1 preBötC neurons do, influence motor pattern and behavioral state, they certainly comprise the inspiratory core oscillator. Two key challenges going forward will be, first, to quantify the proportion of the rhythmogenic preBötC core that is non-Dbx1-derived, and second, to discriminate either on the basis of genetic or other markers, rhythmogenic from non-rhythmogenic Dbx1 neurons.

**Table:** Table 1. Summary of statistics from figures

	Figure	Data structure	Type of test	*p* value
a	1*C*	Undefined	Mann–Whitney *U* test (*n*_1_ = 8, *n*_2_ = 3)	0.286
b	1*C*	Undefined	Mann–Whitney *U* test (*n*_1_ = 8, *n*_2_ = 4)	0.2321
c	3*B*	Normally distributed	Student’s *t* test (*n* = 6)	0.0499
d	3*B*	Normally distributed	Student’s *t* test (*n* = 6)	0.0684
e	3*B*	Normally distributed	Student’s *t* test (*n* = 6)	0.0126
f	3*B*	Normally distributed	Student’s *t* test (*n* = 6)	0.0006
g	3*B*	Normally distributed	Student’s *t* test (*n* = 6)	0.0003
h	3*B*	Normally distributed	Student’s *t* test (*n* = 6)	0.0379
i	3*B*	Normally distributed	Student’s *t* test (*n* = 6)	0.0236
j	3*B*	Normally distributed	Student’s *t* test (*n* = 6)	0.0190, 0.0177
k	3*D*	Normally distributed	Student’s *t* test (*n* = 5)	0.0594
l	3*D*	Normally distributed	Student’s *t* test (*n* = 5)	0.0611
m	3*D*	Normally distributed	Student’s *t* test (*n* = 5)	0.0361
n	3*D*	Normally distributed	Student’s *t* test (*n* = 5)	0.0015
o	3*D*	Normally distributed	Student’s *t* test (*n* = 5)	0.0207
p	3*D*	Normally distributed	Student’s *t* test (*n* = 5)	0.0206
q	3*D*	Normally distributed	Student’s *t* test (*n* = 5)	0.0360
r	3*D*	Normally distributed	Student’s *t* test (*n* = 5)	0.2610
s	3*D*	Normally distributed	Student’s *t* test (*n* = 5)	0.0873
t	5*A_1_*	Normally distributed	Tukey’s HSD (*n* = 4)	1e-6
u	5*A_2_*	Normally distributed	Tukey’s HSD (*n* = 4)	1e-6
v	5*A_1_*	Normally distributed	Tukey’s HSD (*n* = 4)	0.006
w	5*A_2_*	Normally distributed	Tukey’s HSD (*n* = 4)	1e-6
x	6*A_2_*	Normally distributed	Tukey’s HSD (*n* = 4)	0.00217
y	6*A_2_*	Normally distributed	Tukey’s HSD (*n* = 4)	0.0173
z	5*B_1_*	Normally distributed	Tukey’s HSD (*n* = 4)	1e-5
aa	5*B_1_*	Normally distributed	Tukey’s HSD (*n* = 4)	1e-6
bb	5*B_1_*	Normally distributed	Tukey’s HSD (*n* = 4)	4e-5
cc	5*B_1_*	Normally distributed	Tukey’s HSD (*n* = 4)	1e-5
dd	7*B*	Normally distributed	Student’s *t* test (*n* = 4)	0.0273
ee	7*B*	Normally distributed	Student’s *t* test (*n* = 4)	0.0048
ff	7*B*	Normally distributed	Student’s *t* test (*n* = 4)	0.0273
gg	7*D*	Normally distributed	Student’s *t* test (*n* = 4)	0.0389
hh	9*A_1_*	Normally distributed	Tukey’s HSD (*n* = 4)	1e-6
ii	9*A_2_*	Normally distributed	Tukey’s HSD (*n* = 4)	1e-6
jj	9*A_1_*	Normally distributed	Tukey’s HSD (*n* = 4)	1e-6
kk	9*B_1_*	Normally distributed	Tukey’s HSD (*n* = 4)	1e-6
ll	9*B_1_*	Normally distributed	Tukey’s HSD (*n* = 4)	1e-5
mm	9*B_1_*	Normally distributed	Tukey’s HSD (*n* = 4)	0.0499
nn	9*B_2_*	Normally distributed	Tukey’s HSD (*n* = 4)	1e-6

## Synthesis

Reviewing Editor: Muriel Thoby-Brisson, CNRS UMR 5287 Université Bordeaux 2 Decisions are customarily a result of the Reviewing Editor and the peer reviewers coming together and discussing their recommendations until a consensus is reached. When revisions are invited, a fact-based synthesis statement explaining their decision and outlining what is needed to prepare a revision will be listed below. The following reviewer(s) agreed to reveal their identity: Jan-Marino Ramirez, Hidehiko Koizumi

The two reviewers and myself acknowledge the excellence and elegance of the study you decided to submit to eNeuro. While we all agree on the fact that it confirms the primordial role of Dbx excitatory neurons in respiratory rhythmogenesis it nevertheless brings additional findings in more physiological conditions showing Dbx1 neurons involvement in unanesthetized adult mice with intact sensorimotor feedback.

Despite the fact that the authors addressed most of the concerns of the previous round of revisions, a few minor points remain to be at least discussed:

1- The first concern is about the potential role of Dbx1-derived glia in respiratory rhythmogenesis and their possible activation in AchT optogenetic experiments.

The specific comment is: The Reviewer maintains his contention that Dbx1-derived glia cells may be involved in controlling the respiratory rhythm. Possible involvement of glia cells in the results obtained in the ArchT mice experiments could not be excluded, even though their contribution to respiratory rhythm generation has not been well defined in the field.

As the authors stated that ArchT expression in glia cells would be ~40%, but Catch expression is completely limited to neurons. This difference may explain the experimental results that only the highest laser intensity showed significant increase of breathing frequency (f) in CatCh Awake mice. In general, the laser effects in CatCh mice were less significant than those in ArchT mice, probably because of no contribution of glia cells in CatCh mice compared to ArchT mice, which has contribution of glia cells?

Please discuss this issue more carefully in the Discussion section.

2- The second concern is the following :

We do not know how much membrane depolarization is induced by each laser power in CatCh-expressing Dbx1 neurons, while we know it in ArchT expressing neurons (Fig. 1). How did the authors choose the laser intensity of 6.8, 8.6, 10.2 mW for CatCh mice, which is the same laser power as for ArchT mice? It is critical to provide some evidence that the membrane potential perturbations induced by these laser powers are within the normal physiological ranges. For example, 10.2 mW laser would induce the membrane potential depolarization far beyond the normal physiological value?

After discussion the reviewers agree that this might be difficult to assess experimentally and providing some kind of evidence, possibly based on literature would be sufficient.

3. Why unilateral laser illumination in CatCh experiments, but bilateral laser illumination in ArchT experiments? Because of technical difficulty? By applying laser illumination bilaterally in CatCh experiment, would we see significant effects also in lower laser power?

4. Finally to more clearly state the study, one reviewer suggests to change the title to “Dbx1 pre-Bötzinger complex interneurons comprise the core inspiratory oscillator for breathing in unanesthetized adult mice”.
